# Lactate cross-talk in host–pathogen interactions

**DOI:** 10.1042/BCJ20210263

**Published:** 2021-09-07

**Authors:** Alba Llibre, Frances S. Grudzinska, Matthew K. O'Shea, Darragh Duffy, David R. Thickett, Claudio Mauro, Aaron Scott

**Affiliations:** 1 Institute of Inflammation and Ageing, College of Medical and Dental Sciences, University of Birmingham, Birmingham, U.K.; 2 Translational Immunology Laboratory, Institut Pasteur, Paris, France; 3 Department of Infection, University Hospitals Birmingham NHS Foundation Trust, Birmingham, U.K.; 4 Institute of Immunology and Immunotherapy, University of Birmingham, Birmingham, U.K.

**Keywords:** host–pathogen interactions, immunometabolism, infection, lactate

## Abstract

Lactate is the main product generated at the end of anaerobic glycolysis or during the Warburg effect and its role as an active signalling molecule is increasingly recognised. Lactate can be released and used by host cells, by pathogens and commensal organisms, thus being essential for the homeostasis of host–microbe interactions. Infection can alter this intricate balance, and the presence of lactate transporters in most human cells including immune cells, as well as in a variety of pathogens (including bacteria, fungi and complex parasites) demonstrates the importance of this metabolite in regulating host–pathogen interactions. This review will cover lactate secretion and sensing in humans and microbes, and will discuss the existing evidence supporting a role for lactate in pathogen growth and persistence, together with lactate's ability to impact the orchestration of effective immune responses. The ubiquitous presence of lactate in the context of infection and the ability of both host cells and pathogens to sense and respond to it, makes manipulation of lactate a potential novel therapeutic strategy. Here, we will discuss the preliminary research that has been carried out in the context of cancer, autoimmunity and inflammation.

## Introduction

### Lactate and its production within the human body

Lactic acid (C_3_H_6_O_3_) was first reported in sour milk in 1780 [1]. Lactate is the anion form of lactic acid and the main product generated at the end of anaerobic glycolysis [2,3] as well as of aerobic glycolysis in highly proliferative cells, also known as the Warburg effect [4]. In this cytosolic central metabolic pathway, one glucose molecule is broken down into two pyruvate molecules, which are converted into lactate by lactate dehydrogenase (LDH). In this process, ATP and NADH molecules are generated, creating usable energy for the cell. Lactate comprises two stereoisomers, d(−) lactate and l(+) lactate, which are metabolised by d-LDH and l-LDH, respectively [5]. Microbial species contain both LDH forms and can, therefore, produce both lactate isoforms, whereas in vertebrates l-LDH and consequently l-lactate prevail [6,7]. Lactate was originally considered a by-product of cell metabolism, however, there is increasing evidence that it can act as a signalling molecule impacting cell behaviour and function [8,9].

Within the human body, lactate is predominantly produced by muscle cells, erythrocytes and the brain, although most tissues are capable of lactate production under anaerobic conditions.

Lactate is present in the blood and tissues in healthy conditions at low concentrations (typically <2 mM) [10]. Pathological levels of lactate were first demonstrated in 1843 in the context of sepsis and haemorrhagic shock [11]; however, the association between hyperlactatemia (>4 mM) and poor outcomes in human disease was not described until 1964 [12].

Today, lactate is routinely measured and used as a biomarker for disease severity in critically ill patients [13]. However, clinical trials targeting clearance of lactate as a marker of resuscitation in sepsis have failed to demonstrate any benefit [14]. The aetiology of hyperlactataemia is mixed and dependent on individual circumstances in body compartments. Hypoxia and hypoperfusion (decreased blood flow) are the main causes of increased lactate, other contributing factors being hypermetabolism, and mitochondrial or liver dysfunction which impact clearance of lactate [15]. Tissue lactate concentrations are often many folds higher than blood lactate, a scenario seen in cancer and inflammatory settings where blood lactate is frequently normal, but tissue lactate may reach 15–40 mM [16–19] (Table 1). These tissue lactate concentrations in solid tumours were measured using mass spectrometry [16] and imaging bioluminescence (measurements in µmol/g) [19], followed by conversion to mM considering the tumour water content [18].

**Table 1.** The estimated range of lactate levels in healthy and pathological human tissues

Disease state	Lactate level (mM)	Reference
Healthy tissue/blood	<2	[10]
Empyema (human)	13.8	[20]
Inflammatory bowel disease (quiescent) (human)	4	[21]
Inflammatory bowel disease (active)	>10	[21]
Bacterial meningitis (human)	13.6	[22]
Murine *S. typhimurium* infected gut lumen	11.7	[23]
Female genital tract in health	6	[24]
Burn wound exudate	3.19	[25]
Cystic fibrosis sputum (colonised but not exacerbating)	9	[26]
Blood derived macrophages infected with *M.tb*	0.56–6.7	[27]
Saliva	0.3–1.3	[28]
Tumour tissue	30–40	[16,17]

The human body is a complex ecosystem, comprised of both human cells and microbial organisms, such as bacteria, viruses and fungi. Many of these commensals produce lactate as part of their life cycle. Important species include lactobacilli, bifidobacteria and enterococci [29,30]. Hence, we harbour lactate-producing bacteria, but also lactate-utilising bacteria [29,31]. Therefore, lactate can be produced and used by both human and commensal cells, creating a complex but necessary network for the homeostasis of host–microbe interactions. This delicate balance can be perturbed by infections, with invading pathogens also having the capacity to secrete, sense and use lactate [32].

### Lactate transporters and receptors

Lactate stereoselective transport across plasma membranes is mediated by six described solute carrier transporters that perform proton-lactate symport (monocarboxylate transporters: SLC16A1, SLC16A7, SLC16A8 and SLC16A3, also known as MCT1–4) and sodium-dependent transporters (SLC5A8 and SLC5A12, also known as SMCT1–2) [33,34]. Structurally, these transporters contain two six-helix bundles, which constitute their transmembrane domain [35]. Each transporter prefers import or export of lactate; however, the transport direction of both systems depends on the lactate gradient, favouring lactate import when extracellular lactate is high, such as in inflamed tissues. These transporters display extensive substrate specificity and also transport alternative monocarboxylic metabolites such as pyruvate or ketone bodies [34]. They differ in their expression pattern and lactate affinity, SLC16A7 (MCT2) has the highest affinity for lactate (*K*_m_ ∼ 0.7 mM) [35] and is predominantly found in liver, kidney, testis and brain [34]. SLC16A8 (MCT3) is restricted to retinal pigment and choroid plexus epithelia, whereas high expression of SLC16A3 (MCT4) is observed in anaerobic and highly glycolytic tissues. SLC5A8 (SMCT1; high affinity) and SLC5A12 (SMCT2; low affinity) expression is enriched in the kidney, and present to a lesser extent in other body tissues such as the intestine, salivary and thyroid glands, brain and retina, and in small intestine and skeletal muscle, respectively [33,36–38]. Inhibitors targeting MCT1-4 transporters currently exist [39,40] and offer great therapeutic potential, as it will be discussed in the *Therapeutics* section.

Importantly, G-protein-coupled receptors (GPR) have been described for lactate. These include GPR81 (HCAR1), which plays an active role in adipose tissue, muscle and brain [41–43], as well as GPR132, which is highly expressed in blood and immune cells, particularly macrophages, and promotes the M2-like phenotype [44–49].

Multiple lactate transporters are expressed in T cells and macrophages [50]. SCL16A1 and SLC16A3 (MCT1 and MCT4) have been identified in macrophages; SLC16A1 (MCT1) has also been described in the CD8+ T cell compartment, whereas CD4+ T cells mainly express SLC5A12 upon activation [8]. Activation of peripheral blood monocyte cells (PBMCs) via stimulation of CD3 causes indirect up-regulation of SLC5A12 in CD14+ monocytes, and in CD19+ B cells at a lower level [51]. Furthermore, CD20+ B cells and CD68+ macrophages were confirmed to express SLC5A12 in inflamed tonsils [51].

Bone marrow-derived neutrophils predominantly express SLC16A3 (MCT4) and have low levels of SLC16A1 (MCT1) and GPR81 [52], with lipopolysaccharide (LPS) activation or bacterial infection increasing expression of SLC16A3 [52]. Peripheral blood neutrophils also express SLC16A1 and SLC16A3 (MCT1 and 4) [53,54]. Antigen-presenting cells such as dendritic cells (DCs) and macrophages have been shown to express GPR81 [55–57] (Table 2).

**Table 2.** Expression of lactate transporters in human immune cells and pathogens

Cell type	Lactate transporter	Reference
Macrophages	GPR132 GPR81 MCT1, MCT4 SLC5A12 (activated)	[44] [56] [51]
CD8+ T cells	MCT1	[8]
CD4+ T Cells	SLC5A12 (activated)	[8]
B cells	SLC5A12 (activated)	[51]
Dendritic cells	GPR81	[55] [57]
Neutrophils	MCT1 MCT4 GPR81	[52,53,58]
Bacteria	Lactate permeases (lctP) (Gonococcus, Haemophillus and Lactic acid bacteria as examples) Aquaglyceroporins (Lactobacillales)	[28,59,60] [61]
Fungi	Jen transporters Gpr1	[62–65]
Parasites	FNT Aquaglyceroporins TbPT0	[66] (*P. falciparum*) [67] (*T. gondii*) [68] (*T. brucei*), [69] (*T. brucei*)

### The role of lactate in the context of immunity

The combination of these lactate transporters and receptors in immune cells ensures a sensing capacity to a changing environment, such as in response to infection and inflammation. This enables the adaptation of immune cells to fluctuating environments, and ultimately permits the orchestration of an appropriate and effective immune response.

In bacteria, lactic acid and sodium lactate diffusion can be facilitated by different transporters (Table 2), including aquaglyceroporins, such as GlpF1 and GlpF4 [61], and lactate permeases, such as LctP [59]. Therefore, bacteria have evolved their own means for the import/export of lactic acid and/or sodium lactate, through a different mechanism to eukaryotes and often regulated by operons. The lactate–proton symporter Jen1 was first identified in *Saccharomyces cerevisiae* [62], and its ortholog has been reported in *Candida* species [63] (Table 2). They present a more diverse repertoire of lactate transporters, including the evolutionarily conserved receptor Gpr1 [64], which is essential for pathogen survival in certain host niches [65].

There has been relatively little investigation of how lactate impacts immune cell function in infection; however; there is a significant body of evidence of the diverse roles lactate plays in both cancer and inflammatory conditions [50]. All three conditions — infection, cancer and inflammation — are characterised by high lactate levels, and they frequently result in hypoxic and acidic environments [8,16,21]. The diverse roles of lactate in cancer and inflammatory settings have been well reviewed [50]. In brief, the impact of lactate varies according to cell type and disease state. Particularly, effector functions and cytotoxicity are altered by lactate in T cells, macrophages, mast and epithelial cells [9]. In addition to cancer cells [4], stromal and immune cells also contribute to the establishment of a lactate-rich environment within tumours [70]. These conditions suppress immune responses and hence promote tumour growth [9].

The impact of lactate in the tumour microenvironment is outside the scope of this review and has been recently covered [9]. However, valuable lessons can be learned from the cancer field in terms of lactate's immunosuppressive function, which could be translated to hyperlactatemia in the context of infection and inflammation.

Both anti- and proinflammatory responses have been described in response to lactate [9]. Lactate can polarise immune cells to both tolerance states to aid immune evasion, and inflammatory phenotypes, which lead to persistence of inflammation. In cancer cells, an association between GPR81 signalling and up-regulation of PD-L1 was shown, resulting in reduced T cell effector function and proliferation [71]. Although most cancer literature points towards a suppression of immune cell function caused by lactate, evidence also exists for lactate enhancing CD8+ T cell cytotoxic capacity and delaying tumour growth [72]. In the myeloid compartment, high lactate concentrations reduce TNF secretion by human monocytes [73], while promoting M2 polarisation in tumour-associated macrophages [16]. Neutrophils are the first responders to inflammation, they are highly glycolytic and contribute to the accumulation of lactate during infection [52]. Murine studies of bone marrow-derived neutrophils have demonstrated that both LPS and *Salmonella. typhimurium* infection induce glycolysis in neutrophils which then rapidly accumulate lactate. Mobilisation of neutrophils from the bone marrow is dependent on GPR81 signalling, and administration of sodium lactate results in recruitment of bone marrow neutrophils to peripheral blood and peritoneum suggesting potential for a positive feedback loop for lactate mediated neutrophil recruitment [52].

This review focuses on the role of lactate in host–pathogen interactions. We will discuss lactate release from human and pathogenic sources. We will examine how lactate impacts current anti-microbial treatments, and how targeting lactate sensing could be used therapeutically to influence infection outcomes.

## Lactate secretion

### In health and disease

In healthy conditions, lactate levels are usually <2 mM in blood and other bodily fluids [10]. Hyperlactataemia during infection results as a complex interplay of tissue hypoxia, enhanced lactate production by activated immune cells, and generation of lactate by both pathogenic and commensal bacteria, combined with reduced clearance of lactate due to hypoperfusion and impaired lactate shuttles [74]. Production of lactate by the human host varies significantly by cell type but is also heavily influenced by environmental factors. In the context of host–pathogen interactions, lactate secretion by immune cells varies hugely. Neutrophils are the most abundant leukocyte and are the first responders to infection. They heavily rely on glycolysis [75,76], while other immune cells have a more flexible metabolism depending on their environment and activation state [77]. Untangling whether lactate is host or pathogen derived is challenging. Many bacteria can produce and utilise both d- and l-lactate, whereas humans generally only produce and utilise l-lactate. In humans, only l-lactate is routinely measured in clinical practice, as d-lactate rarely reaches sufficient levels to cause disease [6].

### Lactic acid producing and utilising bacteria

Lactic acid-producing bacteria (LAB) are the archetypal lactate producers and are the predominant contributors to gut lumen lactate levels [55,78]. LAB have long been recognised to promote host immunity [79] both in the gut mucosa and systemically; however, the mechanisms of this relationship remain to be elucidated. Whilst LAB are not frequently pathogenic in humans, important human pathogens such as *Streptococcus* and *Enterobacteriaceae* are included in this group [80].

In healthy individuals, lactate levels in the faeces are low, despite an abundance of LAB. The major lactate utilisers in the gut are beneficial *Clostridia* species [29]. *Clostridia* produce butyrate, the major fuel for colonocytes and in the absence of butyrate, colonocytes switch from fatty acid metabolism to glucose metabolism, increasing lactate in the gut lumen [32]. Loss of *Clostridia* species is associated with the use of antibiotics as well as infection with enteric pathogens such as *Salmonella enterica typhimurium* (*S. typhimurium*). Pathogens can take advantage of this dysbiosis and use the increased lactate to support their expansion [32], further driving inflammation and lactate generation by host and pathogen. This is also seen in the female genital tract, which is also colonised by LAB [81]; dysbiosis of lactate utilising and producing strains in this body niche enables expansion of pathogens such as *Neisseria gonorrhoea* [82].

### Pathogens

Non-LAB bacteria are also capable of generating lactate as a product of their metabolism by fermentation of glucose or anaerobic respiration. Particularly notable bacteria capable of lactate production are *Staphylococcus aureus*, *Enterobacteria* and the LAB discussed above, although many more human pathogens are capable of generating lactate [83], as it will be detailed in the section *Lactate in non-bacterial pathogens*. In bacteria, lactate transport is controlled by lactate permease (LctP), a symporter that forms an operon with the LDH genes and is highly conserved across lactate utilising species [84].

The source of lactate at tissue sites is likely to vary depending on the degree of bacterial load and infiltration of immune cells, in addition to oxygen tension and availability of nutrients. For example, in an empyema, lactate levels are typically 13 mM [20] and similar levels are also seen in cerebrospinal fluid (CSF) from patients with bacterial meningitis [22]. In these infections pleural fluid and CSF have significant neutrophilic infiltration and high bacterial burden, contributing to lactate production. These conditions — elevated neutrophil count and bacterial numbers — are also seen in sputum samples from patients with cystic fibrosis and respiratory infections where lactate levels correlate with neutrophil burden [26,85].

## Lactate in bacterial pathogens

### Mechanisms of lactate influence on microbial pathogenicity

**Figure 1.**
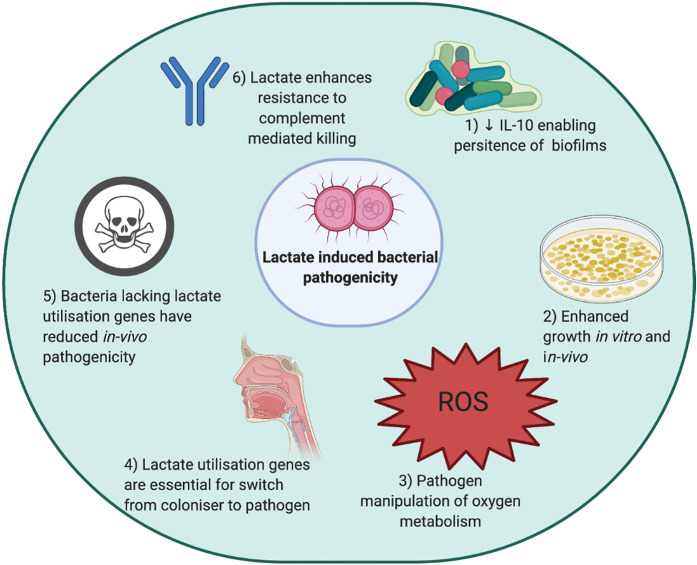
Mechanisms of lactate-driven bacterial pathogenesis. Bacterial pathogens have been demonstrated to use lactate in multiple ways to enhance their pathogenicity. (1) *S. aureus* generated lactate polarises innate immune cells to an immunotolerant state allowing persistence of biofilms. (2) Lactate can be used as a sole carbon source by a variety of bacteria, or bacteria can use lactate as fuel to enhance growth [32,86]. Enhanced growth is seen in Pseudomonas and other bacteria. (3) Reactive oxygen species are a key bactericidal mechanism used by innate immune cells. Lactate enables bacteria to manipulate oxygen metabolism and thus evade killing, a key mechanism for the persistence of *S. aureus* [87]. (4) Many bacteria including *N. meningitidis* and *S. aureus* colonise the nasopharynx, and the ability to invade is crucial in the move to becoming pathogenic. Lactate utilisation genes have been demonstrated to be required for this step [88]. (5) Bacteria lacking lactate utilisation genes have reduced *in vivo* pathogenicity in a variety of animal models of infection, this has been shown for Neisseria species and *H. influenzae* [89]. (6) Complement-mediated killing is reduced in the presence of lactate, mediated by lactate permease [90]. Created using *Biorender.com*.

The mechanisms by which lactate can be used to enhance microbial pathogenicity, including its impact on immune surveillance and pathogen growth/survival, the manipulation of oxygen metabolism, the switch from coloniser to invader pathogen and the resistance to complement-mediated killing are outlined in Figure 1.

**Figure 2.**
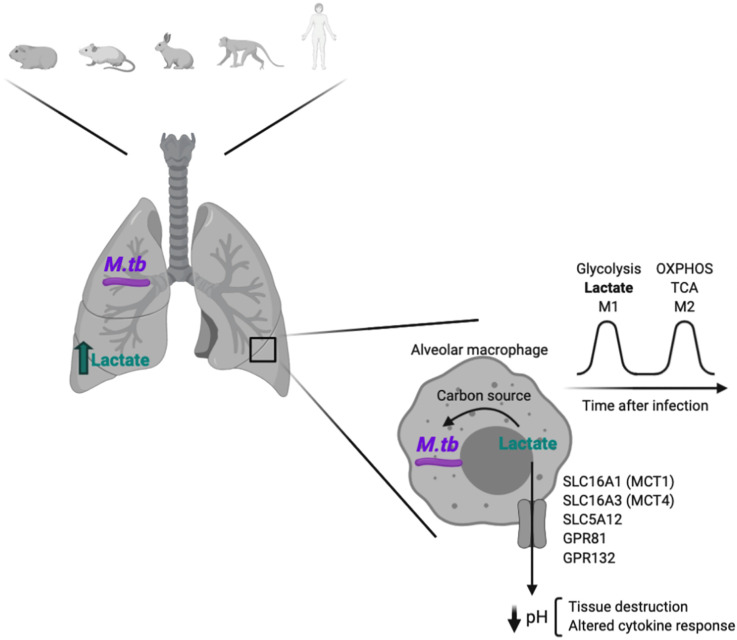
Glycolysis is increased during *Mycobacterium tuberculosis* infection. Glycolysis is induced in the lungs of *Mycobacterium tuberculosis* (*M.tb*) infected hosts, which results in lactate secretion. This increased in glycolysis upon *M.tb* infection has been shown in the lungs of guinea pigs, mice, rabbits, non-human primates and humans. Alveolar macrophages are the first *M.tb* cellular target and, inside them, lactate can be used by *M.tb* as a carbon source, enhancing pathogen survival and cell growth. Lactate can also be exported through different specific transporters, which results in the acidification of the extracellular milieu. This can promote an altered cytokine response as well as tissue destruction. Created using *Biorender.com*.

*Mycobacterium tuberculosis* (*M.tb*) infection will be used as a specific example to illustrate how infection-induced host-derived lactate can change the extracellular milieu, impacting the pathogen's ability to survive and immune function (Figure 2).

### *Mycobacterium tuberculosis* infection

Lactate's role in manipulating both host and pathogen is exemplified by *M.tb* infection. The first target of *M.tb* is the alveolar macrophage (AM) [91]. Upon infection, macrophages switch their metabolism to glycolysis and pyruvate is reduced to lactate [92,93]. Lactate release from macrophages, stimulated with live H37Rv at 10 : 1 multiplicity of infection (MOI) for 24 h, was measured using a fluorescent coupled enzymatic assay [92]. Lactate secretion was also shown to be increased when human monocyte-derived macrophages (hMDMs) and AMs were infected with the live strains of *M.tb* H37Ra and H37Rv for 24 h [93]. In this case, macrophages were infected at MOI 1–5 bacilli/cell for 3 h and then extracellular bacteria were removed. This metabolic shift was shown to be required for host control of H37Ra *M.tb* replication, with CFU/ml measured 72 h post-infection.

Using galactose instead of glucose reduced the ability of the macrophage to control infection in hMDMs and hAM and 2-deoxyglucose had the same effect on mBMDM [93].

The importance of glycolysis in infection resolution has also been shown in an elegant comparison between AMs (which mainly rely on fatty acid oxidation) and interstitial macrophages (predominantly glycolytic), and their respective capacities to control bacterial burden [94]. C57BL/6 mice were intranasally inoculated with the Erdman *M.tb* strain at high CFUs (∼1000).

Interestingly, extracellular flux analysis after 24 h infection of hMDM with H37Rv *M.tb* using MOI 5 : 1 promoted a general metabolic quiescent state, including decreased glycolysis [95]. This was further demonstrated using radiolabelled ^13^C-glucose and showing diminished ^13^C incorporation into several glycolytic intermediates [95]. Dead *M.tb* or Bacillus Calmette-Guerin (BCG) infections induced a glycolytic phenotype, suggesting that *M.tb* has evolved strategies to counteract this metabolic shift. Hackett *et al.* also showed that expression of glycolytic genes was increased when mBMDM were stimulated with heat-killed *M.tb* compared with live *M.tb* H37Ra, and that lactate secretion was also higher on mBMDMs and hMDMs treated with heat-killed *M.tb* compared with live *M.tb* (H37Ra/H37Rv and H37Ra, respectively). They also demonstrated that upon *M.tb* infection, microRNA-21 targets PFK-M, restricting host glycolysis [96].

The current literature assessing the metabolic impact of *M.tb* infection in macrophages uses a variety of models and therefore drawing conclusive answers is challenging. Some of the reported differences are due to the use of murine versus hMDMs following different differentiation protocols, to different strains of live *M.tb*, dead *M.tb* or *M.tb* lysates, different MOIs and/or route and duration of infection.

A biphasic metabolic response for the infected macrophage has been proposed [97], based on transcriptomic analysis of published datasets, including C57BL/6 BMDMs infected with *M.tb* H37Rv [98], B6D2F1 BMDMs infected with *M.tb* CDC1551 or HN878 [99] and BALB/c BMDMs infected with *M.tb* HN878 [100]. This model proposes a first phase (4–8 h post-infection) characterised by a switch to glycolysis, which is accompanied by HIF-1a expression, enabling M1 polarisation and the orchestration of an effective antimicrobial programme, including the production of pro-inflammatory effector molecules. In the second phase (24–48 h post-infection), macrophages transition towards mitochondrial oxidative metabolism with high TCA activity. The dampening of M1 polarisation allows increased *M.tb* survival and growth [97,101]. According to this biphasic model, important metabolic changes might be happening by ∼24 h post-infection. It is, therefore, possible that measurements of lactate at this time point reflect previous macrophage engagement in glycolysis [92,93], while the strategies of *M.tb* to supress glycolysis are already in place 24 h post-infection [95]. It is also possible that glycolytic genes are up-regulated at the transcript level, but *M.tb* has strategies in place to supress glycolysis further down the line (i.e. at the protein level as shown by Hackett *et al.* [96]. In that case, transcriptional up-regulation of glycolytic genes and decreased glycolysis measured through extracellular flux analyses would not be incompatible. Also, extracellular flux studies [95] oppose the increased TCA activity 24 h post-infection showed using transcriptomic analysis [97].

RNA-sequencing analysis of *M.tb* H37Rv-infected mice demonstrated up-regulation of glucose transporters and glycolytic enzymes, including *Gapdh*, *Hk3* and *Ldha* in lung tissue [102]. They also showed increased levels of HK3 and LDHA protein through immunohistochemical staining of *M.tb* infected mouse lungs. These two glycolytic enzymes partially co-localised with macrophages (IBA-1+) and T cells (CD3+). Using a rabbit aerosol infection model with *M.tb* HN878, Subbian and colleagues [103] showed up-regulation of gene transcripts related to the Warburg effect between 4 and 12 weeks post-infection, including some glucose transporters, glycolytic enzymes and monocarboxylate transporters [104]. Transcriptomics of lung granulomas from patients with active TB expressed increased Warburg-effect related genes in comparison with non-granulomatous portions of the same lung [104,105]. Furthermore, a recent study using a dual RNA-seq approach to investigate gene expression of both *M.tb* and the human host described a shift towards host glycolysis in TB sputa, in comparison with non-TB controls [106]. Thus, both at the transcript and protein levels and using different *in vivo* infection models and human data, the evidence points towards an increase in glycolysis in the lungs of *M.tb* infected hosts.

Further evidence of this metabolic shift towards glycolysis comes from lactate measurements. Mice infected with *M.tb* H37Rv via aerosols showed increased lactate in their lungs 4 weeks post-infection, measured by nuclear magnetic resonance (NMR) [107]. Similar findings were reported in a guinea pig model of infection also infected with *M.tb* H37Rv and using NMR [108]. Here, up-regulation of lactate was described as infection progressed (from day 15 onwards) in granulomatous tissues. High-resolution mass spectrometry has been used to measure the mouse lung metabolome infected with *M.tb* H37Rv intratracheally. In this case, only marginal and moderate lactate increase was detected at 4- and 9-weeks post-infection, respectively [109]. The different infection route, as well as the dose of *M.tb* given (10-fold higher in the latter), may account for some of the observed differences. Interestingly, enhanced LDH levels were detected in serum and bronchoalveolar lavage (BAL) from TB patients [110,111].

The impact of lactate sensing on immune cell function in the context of *M.tb* infection has been limitedly studied. Preliminary studies suggest the enhanced killing of *M.tb* by human macrophages incubated with sodium l-lactate [112]. Enhanced lactate secretion also lowers tissue pH, and extracellular acidosis has been shown to promote tissue destruction and altered cytokine secretion, impacting the control of infection and patient survival in tuberculosis [113,114].

*M.tb* is also able to use lactate as a sole carbon source [115], and when actively replicating it is able to use lactate or pyruvate more efficiently than glucose [27]. *M.tb* has a preference for pyruvate as carbon source, up-regulation of LDHA in infected macrophages being a mechanism to prevent pyruvate access to the pathogen (and consequent growth) by converting it to lactate [116]. It has been shown that *M.tb* is able to grow in lactate concentrations of up to 44 mM in *in vitro* cultures [27], however, it has also been described that greater than 20 mM lactate inhibits *M.tb* growth [117]. Further confirming this, deletion of *ldh* (*lldD2*, Rv1872c) in *M.tb* is detrimental to growth in lactate containing media, suggesting potential lactate toxicity when *M.tb* cannot metabolise it. Inside the infected macrophage, lactate concentrations have been estimated to be between 0.56 and 6.7 mM [27]. The observed discrepancies may be explained by strain and/or culture conditions differences [27]. The TB granuloma and the tumour microenvironment share relevant similarities such as nutrients and oxygen gradients potentially leading to hypoxia (a driver of anaerobic glycolysis and lactate release) and nutrient scarcity in certain regions [118–120]. As discussed previously, lung tissue from *M.tb* infected hosts present evidence of high glycolytic rates and lactate production. These factors impact pathogen survival [118,121] as well as immune cell function and infection resolution [112,122]. Furthermore, *M.tb* as an intra-cellular pathogen is commonly exposed to oxidative stress, and it is thought that lactate oxidation also plays a role in protecting *M.tb* from NO· [117] by conversion to pyruvate and consequent NAD+ generation, which helps with redox control.

Genetic studies on *M.tb* strains point towards the presence of lactate in *M.tb* niches.

Both promoter and non-synonymous gene mutations have been identified in genomic analyses of *M.tb* lineage 4 genomes within the *lldD2* gene, encoding for l-lactate dehydrogenase. These mutations evolved independently more than 100 times and emerged in all continents. Furthermore, some of them promoted increased transmissibility [123]. Another study that analysed publicly available genomes from different lineages found evidence of positive selection in the *lldD2* gene [124], suggesting metabolic adaptations to lactate-rich environments. Supporting this same notion, the Colombian clinical isolate UT127 was shown to cope with scarce lipid availability by up-regulating *lldD2* (Rv1872c), suggesting the potential use of lactate as an alternative carbon source [125].

In summary, the evidence converges towards increased glycolysis and lactate release in the context of TB disease, with *M.tb* having developed efficient strategies to dampen this metabolic shift [95,96]. Due to the wide range of models and techniques used, it is still hard to define the precise kinetics of such metabolic rearrangements. Further investigation is also needed to specifically define when and where within the lung lesions glycolysis occurs, whether it happens in aerobic or anaerobic conditions [119], and its consequences in terms of lactate production, immune function and infection resolution. It is also possible that glycolysis and lactate production happen in immune cells other than macrophages during *M.tb* infection. For instance, neutrophilic infiltration is a well-described phenomenon in TB lung lesions [126,127], and neutrophils highly rely on glycolysis [52,75]. Also, switches towards glycolysis have been described in other lung cells including fibroblasts [128] and lung epithelial cells [129].

Lactate, therefore, acts at multiple levels of the host–pathogen interface and further investigation is required to fully elucidate its detrimental or beneficial effects in the ability of the macrophage to clear *M.tb* infection, and potentially identify new host-therapeutic targets [130–132]. There are limited studies exploring the role of lactate in other infection settings and disease states. Next, we discuss the evidence that exists for other organisms and the mechanisms employed.

### The impact of lactate on bacterial persistence in biofilms

In the field of cancer immunometabolism, lactate has been demonstrated to induce immunotolerance and enable cancer cells to evade detection. Similar mechanisms may support the development of chronic infection.

*Staphylococcus aureus* (*S. aureus*) is an important pathogen in human disease and can form biofilms, which are challenging to treat and require prolonged courses of antibiotics, particularly in infections of prosthetic material. Host-derived IL-10 is crucial for the maintenance of the biofilm with anti-inflammatory myeloid-derived suppressor cells and anti-inflammatory macrophages enabling biofilm persistence [133]. Lactate derived from *S. aureus* has been shown to regulate the inflammatory properties of the innate immune response and drive biofilm persistence. In a prosthetic joint infection model, *S. aureus* lacking *ldh* caused less bacterial burden and decreased levels of IL-10 [134]. IL-10 is also a potent suppressor of T cells [135]. The proposed mechanism is that lactate can inhibit histone deacetylase and therefore regulate gene expression in innate immune cells leading to the anti-inflammatory phenotype, high production of IL-10, thereby promoting the persistence of the biofilm. These findings could aid a better understanding of other biofilm infections that cause medical complications, such as *Mycobacterium abcessus* [136,137].

### Lactate promotes enhanced bacterial growth

As seen in *M.tb* lactate can be used as a sole carbon source, or as a combined fuel by many other microorganisms [86,138]. This flexibility can enhance bacterial growth compared with use of glucose alone. d and l-lactate are likely to be present during infection, and *Escherichia coli* (*E. coli*) and *Pseudomonas aeruginosa* can utilise them both [138,139]. Pseudomonas species in the context of biofilms have been demonstrated to cross-feed d-lactate produced by its own fermentation [139].

Within inflammatory compartments caused by infection, lactate is abundant. Several organisms have adapted to utilise lactate, and further drive inflammation to increase local lactate levels to support their expansion. In a murine model of *S. typhimurium* infection lactate reached 11.7 mM in the gut lumen [32]. This is thought to be driven by *S. typhimurium*-induced dysbiosis causing a switch in intestinal epithelial cell metabolism from fatty acid to glucose metabolism. The ability to oxidise lactate by *S. typhimurium* confers a fitness advantage in the gut, as lactate utilisation genes (*lldP*, *lldR* and *lldD*) are rapidly induced upon exposure to l-lactate [23]. The inflammation induced by expansion of *S. typhimurium* drives further dysbiosis and correlates with increased availability of lactate [32].

The female genital tract also has high levels of lactate (∼6 mM) [24]. *Neisseria gonorrhoea* (*N. gonorrhea*) [140] is a common pathogen in the genital tract, that is able to utilise lactate as a sole fuel and can result in significant morbidity [140,141].

The flexibility to use varied carbon sources enables growth and success in different niches, as exemplified by *Neisseria meningitidis* (*N. meningitidis*). *N. meningitidi*s can use lactate as a fuel when glucose is unavailable [24,82]. In bacterial meningitis, CSF glucose levels are initially high but fall rapidly, whereas lactate (13 mM) on average) is replenished, presumably from glycolysis of activated immune cells and bacteria [22]. *N. meningitidis* growth is enhanced when lactate is added to glucose-containing media [142].

The ability to breakdown pyruvate to lactate and NADH has been shown to be beneficial for the growth of *Streptococcus pneumoniae* (*S. pnuemoniae*). *S. pnuemoniae* possesses a single copy of *ldh* [143]. Deletion of *ldh* abolishes lactate production entirely, and *ldh* mutants demonstrated 2.5-fold lower growth when glucose was the sole carbon source [144] compared with media containing glucose and galactose. The *ldh* mutant showed a shift to mixed acid fermentation rather than homolactic fermentation with accumulation of pyruvate [144].

*Pseudomonas aeruginosa* cultured in burn wound exudate rather than LB media demonstrated preferential use of lactate as a carbon source as compared with glucose. Lactate levels in the burn wound exudate were typically 3.19 mM [25] and declined over the 24 h culture period. *Pseudomonas*’ preference for lactate as a carbon source was confirmed in experiments where a cystic fibrosis like sputum media containing 9 mM lactate demonstrated superior growth [26] compared with conventional media.

### Manipulation of oxygen metabolism

Inflammatory nitric oxide (NO·) is a crucial bactericidal mechanism for leukocytes. NO· has a range of cytotoxic effects, however, certain bacterial species, such as *S. aureus* display resistance to NO·. This resistance may play a role in the ability of *S. aureus* to persist and colonise the nasopharynx. Host-derived NO· prevents aerobic respiration by *S. aureus* and induces fermentation of glucose, thereby generating significant levels of l-lactate. l-lactate is then converted to pyruvate generating NAD+, which is used to maintain redox balance in the face of NO· [87]. Additionally, *S. aureus* possesses NAD independent LDH (iLDH) which oxidises lactate to pyruvate with simultaneous reduction of the respiratory quinone pool [145]. Inactivation of *ldh1* impairs *S. aureus* growth in the presence of NO· and, similarly, the *ldh1* mutant *S. aureus* had significantly reduced virulence in a murine model of sepsis. However, in mice lacking the ability to generate NO· the virulence of the *ldh1* mutant was partially restored [87]. This was also replicated in a *S. aureus* mutant lacking iLDH or Lqo [145]. Suggesting that iLDH and LDH in *S. aureus* enables the pathogen to use host or pathogen-derived lactate to protect from exogenous NO·. The bactericidal effect of NO· are clearly abrogated in *S. aureus*, however, it is important to note NO· is also able to influence bacteria by other cytotoxic mechanisms [146] which are not fully explained by this observation.

Competition between species and manipulation of oxygen metabolism has also been shown to enhance the survival of other bacteria. For example, *N. meningitidis* is able to utilise lactate derived from neutrophil glycolysis to enhance oxygen consumption and therefore reduce oxygen availability for bactericidal mechanisms by neutrophils [141].

### Ability to move from coloniser to invasive pathogen

*N. meningitidis* colonises the nasopharynx, and a crucial step in dissemination and its ability to cause invasive disease is the dispersal of micro-colonies, a process during which bacteria detach and access new sites. This has been shown in a nasal mucosa explant cell culture model to be dependent on both d- and l-lactate metabolism [88], as well as in isolated epithelial cells. It is important to consider that in single pathogen *in vitro or ex vivo* models there is invariably simplification of the complex interplay taking place *in vivo* with competition between pathogens and a rapidly changing immune environment, none the less it is increasingly clear that lactate plays an important role in pathogen virulence. A similar effect was seen in a different strain of *N. meningitidis* and *N. gonorrhea* [147]. It was confirmed that expression of lactate utilisation genes (*lctP*) is required for nasopharyngeal colonisation by *N. meningitidis* [88], despite the mutant having normal adhesion.

### Resistance to serum mediated killing

Complement-mediated killing is a crucial host bactericidal mechanism. Lactate is a key virulence factor in protection from complement-mediated killing in *N. meningitidis* as the presence of *lctP* is required for avoidance of serum-based killing [142]. The reduced virulence of the *lctP* mutant strain was restored to normal levels in the absence of serum complement [142]. *N. gonorrhea* has enhanced resistance to serum-based killing mediated by lactate metabolism, leading to enhanced LPS sialylation, independent of sialyltransferase, which prevents the bactericidal action of antibodies and complement [82].

*Haemophilus influenzae* strain b (*Hib*) demonstrates increased resistance to serum mediated killing when grown in either nasopharyngeal aspirate or human serum which contains lactate compared with lactate free culture media [90]. This phenotypic change was inducible by as little as 30 min incubation in human serum. The mechanism underlying this observation is unclear as it was shown that deletion of *lctP* did not affect survival in human serum [28]. However, these effects may be strain dependent as a strain of *lctP* mutated non-typeable *H. influenzae* (NTHi) had impaired growth compared with a wild type strain, but again this was not replicated in a different NTHi strain [28]. Resistance to serum mediated killing could simply relate to a carbon source preference for lactate in *Hib* increasing growth velocity. Again it is important to acknowledge that these are *in vitro* findings when bacteria are cultured at densities exceeding those seen in human and animal infections, with tightly regulated nutrient availability not reflecting true infection states.

### *In vivo* survival

*In vivo* survival is much more complex than the *in vitro* culture described above, and the central role of lactate utilisation has been demonstrated in animal models of infection. Loss of lactate utilisation genes reduces virulence, attenuates bacteria's ability to colonise and results in less severe infection. *N. gonorrhea* can utilise both d and l-lactate, and lactate controls key virulence factors as loss of *ldh* and *lctP* results in decreased survival in cervical epithelial cells under microaerobic conditions [140].

*Ldh* mutant pneumococcus was unable to cause bacteriaemia in a mouse model of sepsis and mice had significantly longer survival compared with infection with wild type strains [144]. *lctP* mutant *N. meningitidis* has an attenuated ability to cause bacteriaemia compared with wild type strains in a rat model [142]. Signature tagged mutagenesis of *H. influenzae* identified l-lactate permease as crucial for *in vivo* survival [89]. *lctP* mutant *N. gonorrhea* has reduced ability to colonise the murine vagina compared with WT [82,148].

## Lactate in non-bacterial pathogens

### Viruses

Viruses are obligate parasites and there is no evidence of direct lactate sensing capacity. However, they often rely on endocytic pathways for cell entry in which a decrease in pH acts as a signal for host penetration [149]. In particular, the requirement for a drop in pH for viral fusion and entry has been described in multiple viruses such as the Semliki forest virus [150] and influenza [151,152]. In Epstein–Barr virus (EBV)-derived lymphomas [153], EBV increases LDH-A expression and, consequently, lactate secretion by B lymphoma cells, resulting in down-regulation of viral microRNA promoting cancer growth. Viruses can also influence cellular processes such as metabolism to impose an environment that favours their replication [154,155]. Adenovirus infection of human cell lines HEK293 and 1G3 resulted in 2-fold and 4-fold increases of glucose consumption and lactate release, respectively [156,157]. Similar trends have been observed with Herpes simplex virus 1 and 2 [158,159], human CMV [158,160,161] and influenza [162–164]. Also, lactate limits retinoic-acid-inducible gene I (RIG-I)-like receptor (RLR) signalling, diminishing type I interferon production and preventing viral clearance [165]. Lactate is directly sensed by the mitochondrial antiviral-signalling (MAVS) protein, blocking MAVS aggregation and downstream RLR signalling. Inactivating LDH-A, therefore, results in increased type I IFN secretion and protection of mice from viral infection. In summary, lactate is closely linked to viral infections, from viruses increasing LDH expression and lactate production, to lactate specifically targeting key proteins in antiviral signalling.

### Fungi

**Figure 3.**
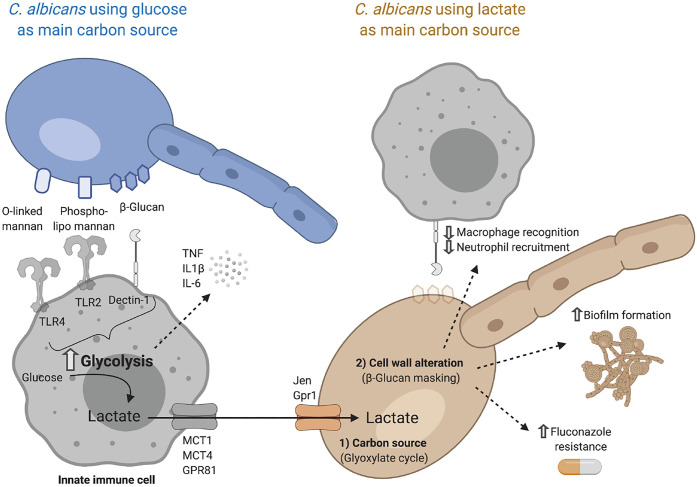
Lactate cross-talk between *Candida albicans* and phagocytic immune cells. The main pattern recognition receptors (PRRs) involved in the recognition of *C. albicans* are C-type lectin receptors (CLRs) and Toll-like receptors (TLRs) expressed on the surface of innate immune cells with high phagocytic capacity (neutrophils, macrophages and dendritic cells). Sensing of *C. albicans* triggers a metabolic shift towards glycolysis, which is essential for the effective production and release of proinflammatory cytokines such as TNF, IL1β and IL-6. Active glycolysis results in the production of lactate, which can be exported through different lactate transporters including MCT1, MCT4 and GPR81, depending on cell type (see Table 2). Once this lactate is in the extracellular milieu *C. albicans* can take it up through the Jen and Gpr1 transporters and use it to thrive in nutrient-restricted body niches. Lactate can not only be used as a carbon source, fuelling metabolic pathways such as the glyoxylate shunt, but it can also alter cell wall composition. These changes largely impact the ability of the host to mount an effective immune response. One of the best-described strategies is the masking of β-glucan, which results in decreased macrophage recognition and neutrophil recruitment. Furthermore, when *C. albicans* uses lactate as the main carbon source, biofilm formation, as well as resistance to antifungal drugs such as fluconazole, are increased. Created using *Biorender.com*.

Recognition of fungal cell wall components triggers glycolysis in innate immune cells [166–169]. This metabolic adaptation has been described in response to *Candida albicans* [166–168], *Aspergillus fumigatus* [170] and *Cryptococcus gatti* [171] infection. The shift to glycolysis enables optimal immune responses through the release of proinflammatory cytokines [166,170]. The Jen transporters, which perform proton-lactate symport, are expressed in fungal species [62,63]. Pathogens such as Candida can use lactate as a carbon source to thrive in nutrient-restricted body niches such as the gut or the vagina [172]. The role of lactate in *C. albicans* infection goes beyond nutrition, as lactate is directly involved in modulating pathogen–phagocyte interactions (Figure 3). Lactate increases biofilm formation, enhances resistance to different host-stressors and diminishes macrophage recognition [64,172,173]. β-glucan, a major cell wall component, can be masked after triggering by lactate which has been described as one of the key immune evasion strategies employed by *C. albicans* [64,174]. *C. albicans* grown with lactate as its main carbon source also impacted immune cell function increasing secretion of the immunosuppressive cytokine IL-10, while decreasing IL-17, which plays a key role in protection against invasive candidiasis [175,176].

### Parasites

Glycolysis is the preferred metabolic pathway for fulfilling the energy requirements of *Plasmodium falciparum*, the intra-erythrocytic parasite that causes malaria [177]. This process generates large quantities of lactate that is primarily expelled through pfFNT, a surface transporter of the formate-nitrite family [66]. Erythrocytes will in turn release lactate to the bloodstream through SLC16A1 (MCT1) [178]. *Toxoplasma gondii* is a major zoonotic pathogen that also relies on glycolysis for its growth, despite being metabolically versatile [179,180]. Several studies have shown how *Toxoplasma* LDH determines bradyzoite (slowly dividing phase) differentiation, virulence and chronic infection [179,181,182]. Whether lactate is directly implicated or whether these findings reflect the insufficiency of oxidative phosphorylation to meet the parasite's energetic requirements is still unknown. In general, the use of and response to lactate in parasites has not been well characterised in detail. However, there is abundant evidence of the evolutionary pressure for all these parasites to have effective lactate transport systems in place.

## Therapeutics

**Figure 4.**
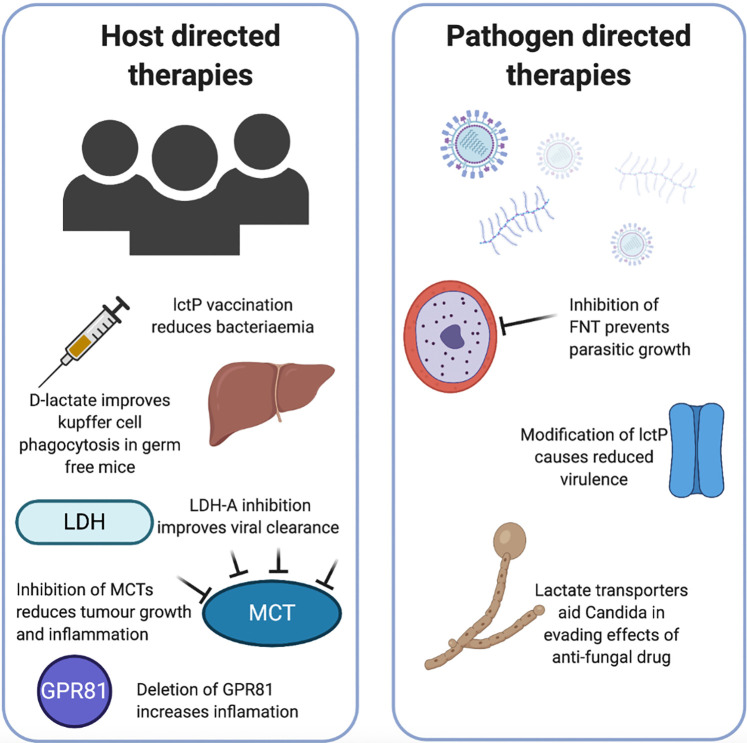
Lactate directed host and pathogen therapeutic avenues. Lactate directed therapeutics can be considered to target host or pathogen utilisation of lactate. In the host, vaccination of mice with recombinant *lctP* was partially protective against *N. meningitidis* bacteraemia. Supplementing germ-free mice with either d-lactate or LAB improved the phagocytic capacity of Kupffer cells in the liver preventing bacteriaemia. Inhibition of lactate utilisation tools such as MCT, LDH or the lactate receptor GPR81 has been demonstrated to improve viral clearance, reduce tumour burden while deletion of GPR81 increases susceptibility to inflammation. For pathogen directed therapies inhibition of lactate transporters prevents growth of plasmodium falciparum, and in other bacteria deletion of lctP causes reduced virulence *in vivo*. Lactate transporters are crucial for Candida to avoid antifungal drugs. LctP, lactate permease; LAB, lactic acid-producing bacteria; MCT, monocarboxylate transporters (lactate transporters in vertebrates) and LDH, lactate dehydrogenase. Created using *Biorender.com*.

Human studies specifically modulating lactate in infection are scarce, however, research has been done regarding the therapeutic potential of targeting lactate in the context of cancer, autoimmunity and inflammation. The most common approaches include blocking specific lactate transporters, targeting LDH or adding lactate (Figure 4).

### Cancer therapeutics

The potential of MCTs and LDH inhibitors for cancer treatment has been reviewed elsewhere [9]. Both small compounds and RNA silencing strategies have been employed with promising results. For instance, the orally dosed MCT inhibitor AZD3695 is undergoing phase I/II trials in lymphoma (NCT017915950). It is promising that modulators of lactate transport are in use in human studies, suggesting that safety and tolerability will soon be established to enable use in other human diseases. Furthermore, the use of shRNAs to knock down LDH-A resulted in decreased proliferation of tumour cells [183].

The use of oral MCT inhibitors in human studies of lymphoma are promising, but care needs to be taken in translating tolerability and safety to infection. Some infections are chronic and associated with immunosuppression, but initial stages of acute infection including sepsis are associated with overwhelming immune activation.

To begin to therapeutically target lactate we need to first understand the role lactate plays in the diverse infection settings in terms of host response, across organ systems and disease states to prevent inadvertent harm. Developing a clearer understanding of pathogens lactate utilisation but also symbionts may allow directed targeting of their specific transporters avoiding harm to the host.

### Autoimmunity and inflammation

The role of lactate as an immune modulator in inflammatory conditions is now well accepted. Studies in a collagen-induced arthritis mouse model showed that silencing of SLC16A3 (MCT4) reduced the severity of arthritis [184]. Also, SLC16A3 (MCT4) transcripts were up-regulated in synovial fibroblasts from rheumatoid arthritis patients compared with osteoarthritis patients, and knockdown of SLC16A3 (MCT4) prevented their proliferation [184]. Furthermore, in other mouse models of arthritis and peritonitis, antibody, or shRNA blockade of SLC5A12 also reduced disease severity, which was accompanied by restored T cell function [8,51].

In sterile models of chemically induced hepatitis and pancreatitis, pre-treatment with lactate reduced inflammation by attenuating TLR induced inflammatory cytokines through GPR81 signalling [185]. The crucial role of lactate sensing by GPR81 was confirmed as deletion of GPR81 increased susceptibility of mice to chemically induced colitis, where GPR81 deficient mice had elevated inflammatory cytokines and up-regulation of inflammatory Th1/Th17 cells [78].

### Targeting lactate in the context of infection

Although the therapeutic potential of targeting lactate has not yet been explored in human clinical trials, evidence is accumulating and promising avenues are being investigated.

### Viral infection

The *in vivo* immunosuppressive action of lactate has been demonstrated: lactate is sensed by mitochondrial antiviral signalling proteins reducing RIG-I-like receptor activation to down-regulate type I interferon production providing an immunotolerant state in which the virus was able to evade immune surveillance [165]. Inhibition of LDHA reduced lactate and promoted viral clearance through up-regulation of type I interferon and other inflammatory cytokines [165]. Thus, targeting lactate could directly aid the orchestration of effective antiviral immune responses.

### Bacterial infection

As discussed in previous sections, some bacteria can use lactate as a carbon source. Therefore, targeting bacterial utilisation of lactate could potentially prevent the expansion of bacterial communities. Lactate utilisation genes are crucial for the virulence of many organisms, and targeting them with vaccination *in vivo*, or by *in vitro* inhibition seems to have some promise [186]. For instance, the expression of lactate permease (*lctP*) by *N. meningitidis* is crucial for its pathogenicity, and *lctP* has been explored for its potential as a vaccine target. Treatment of mice with recombinant *lctP* partially protected them from *N. meningitidis* bacteriaemia [186].

### Parasitic infection

Targeting lactate transport in parasites has also been trialled *in vitro*, where inhibition of formate–nitrate transporters impairs growth and leads to parasitic death in both *P. falciparum* and Toxoplasmosis [67,187]. Targeting lactate transporters in candida species could also offer therapeutic potential since they have been shown to aid *C. albicans* and *C. glabrata* in evading the effects of antifungal drugs [65,172].

Based on the reviewed evidence, a degree of immunosuppression induced by lactate in acute infection may be protective in preventing overwhelming inflammation-causing myocardial and associated organ damage. Alternatively, in chronic infections, the immunosuppressive effect of lactate may be more deleterious by allowing the virus to remain undetected and actively replicate. Further investigations are warranted to fully understand the complex interplay between the pathogen and the host in terms of responses to lactate. Only then we will be able to target lactate and improve infection outcome.

### Beneficial role of lactate in host–pathogen relations

Evidence also exists for the beneficial roles of lactate. Specifically, in the context of bacterial derived lactate, there is a further challenge to be considered. LAB are the main producers of d-lactate in the gut. Dysbiosis occurs commonly in disease states and is associated with increased risk of sepsis and disseminated infection [188]. Giving critically ill patients probiotics containing LAB protects them from nosocomial acquired infection [189]. This link between loss of intestinal LAB and health is clear, but the mechanism that is able to impact distant immune cells remained unclear until recently [190]. The role of intestinal microbiota-derived d-lactate in maintaining crucial immune surveillance distant to the gut was demonstrated. In germ-free mice, the phagocytic capacity of Kupffer cells was significantly reduced, leading to the persistence of *S. aureus* bacteriaemia. Supplementing their diet with d-lactate partially restored the ability to clear bacteriaemia, and the same effect was demonstrated when gnotobiotic mice were supplemented with specific LAB which produced high levels of d-lactate. d-lactate levels were shown to be elevated in the gut, and portal vein, but not systemically. It is also important to note that although d-lactate played a crucial role, the phagocytic capacity was not fully restored, likely reflecting the contribution of other metabolites [190]. This study opens new avenues for using lactate itself as a therapeutic molecule.

Further characterisation of lactate utilisation in both symbiotic and pathogenic organisms, and the role of lactate on influencing host immunity during acute and chronic infection, will allow us to understand this complex interplay. Thus, it seems likely that manipulation of lactate in infection, whether host or pathogen derived, will require a balance between influencing pathogenic organisms, while enhancing the beneficial relationship with symbionts.

## Conclusion

In recent years, we have seen our understanding of lactate evolve from a mere by-product of cellular metabolism to an active signalling molecule playing an active role in cancer, inflammation and host–pathogen interactions. Curating a comprehensive view of the consequences of increased lactate concentrations in the context of infection and how this affects both pathogen and immune function is challenging. Lactate can be released by host cells, as well as pathogenic and commensal microbes. The precise impact of lactate on immune cell function seems to be context dependent, as it is mainly immune-suppressive in the tumour microenvironment, while it can favour chronic inflammation in other settings.

Several pathogenic microbial species have evolved mechanisms to use lactate that enhances their pathogenesis. The presence of lactate transporters in most human cells including immune cells, as well as in a wide range of pathogens including bacteria, fungi and complex parasites, is proof of the importance of this metabolite in regulating host and pathogen function in the context of infection. This makes lactate an attractive therapeutic target, and proof of concept studies have already been developed. We still require a deeper understanding of the role lactate plays in the complex interplay between commensals, pathogens, and the host before we can benefit from these potential novel therapeutic approaches.

## Open Access Statement

Open access for this article was enabled by the participation of University of Birmingham in an all-inclusive Read & Publish pilot with Portland Press and the Biochemical Society under a transformative agreement with JISC.

## Abbreviations

AM: alveolar macrophage
BAL: bronchoalveolar lavage
CSF: cerebrospinal fluid
DCs: dendritic cells
EBV: Epstein–Barr virus
GPR: G-protein-coupled receptors
*Hib*: *Haemophilus influenzae* strain b
hMDM: human monocyte-derived macrophage
iLDH: independent LDH
LAB: lactic acid-producing bacteria
LctP: lactate permease
LDH: lactate dehydrogenase
LPS: lipopolysaccharide
MAVS: mitochondrial antiviral-signalling
MOI: multiplicity of infection
NMR: nuclear magnetic resonance
NTHi: non-typeable *H. influenzae*
RLR: retinoic-acid-inducible gene I (RIG-I)-like receptor
